# Suicide after a Diagnosis of Cancer: Follow-Up of 1.4 Million Individuals, 2009–2019

**DOI:** 10.3390/cancers15174315

**Published:** 2023-08-29

**Authors:** Irmina Maria Michalek, Florentino Luciano Caetano dos Santos, Urszula Wojciechowska, Joanna Didkowska

**Affiliations:** Polish National Cancer Registry, Maria Sklodowska-Curie National Research Institute of Oncology, 02-093 Warsaw, Poland

**Keywords:** cancer, suicide, risk, epidemiology, cohort study

## Abstract

**Simple Summary:**

Cancer patients in Poland face a higher risk of suicide. The study focused on over 1 million individuals diagnosed with cancer between 2009 and 2019. Within the first six months of diagnosis, the suicide risk spiked significantly. Cancers of the esophagus, stomach, cervix, and head and neck showed the highest risk. This highlights the urgent need for better mental health support in cancer care, especially during the crucial post-diagnosis period. Detecting signs of suicide risk early on could make a life-saving difference and shape more effective healthcare strategies.

**Abstract:**

**Background:** The study explores whether Polish cancer patients face elevated suicide risk, emphasizing the vital need to comprehend and mitigate their unique mental health struggles. **Methods:** We conducted a cohort study based on Polish National Cancer Registry data (diagnosis in 2009–2019). Age-, sex-, and year-standardized mortality ratios (SMR) are presented with 95% confidence intervals (CIs) overall and by sex. **Results:** The study included 1.43 million individuals diagnosed with cancer. There were 830 suicide cases in this group. The overall SMR for suicide was 1.34 (95% CI 1.25–1.43). The highest risk of suicide death was observed in the first six months after diagnosis (SMR = 1.94, 1.69–2.21): cancers of the heart and pleura (19.15, 2.32–69.18), an unspecified site (3.99, 1.09–10.22), and the esophagus (3.34, 1.08–7.79). The highest overall risk of suicide after cancer diagnosis was observed in esophageal (2.94, 1.47–5.26), gastric (2.70, 2.00–3.57), cervical (2.20, 1.06–4.05), and head and neck cancers (2.06, 1.52–2.72). **Conclusions:** Patients with cancer face significantly higher suicide risk, peaking within six months post-diagnosis. Urgent integration of suicide risk screening and prevention into cancer care is crucial, supporting mental well-being and guiding proactive healthcare strategies.

## 1. Introduction

Cancer is a significant contributor to the global burden of disease, and it is forecast that this burden will continue to grow for at least the next two decades [1]. There is widespread agreement that a cancer diagnosis substantially impacts human mental well-being, possibly inducing suicidal behaviors [2,3]. There is evidence that suicide rates are higher in patients with cancer than in the general population [4,5,6,7]. Even if, compared to other causes of death, the fraction of suicidal deaths among patients with cancer is minimal, the majority of these fatalities are potentially avoidable.

Global concern about the risk of dying by suicide by individuals previously diagnosed with cancer is growing [8,9,10,11,12]. According to a widely referenced British study, patients with cancer had a 20% greater risk of suicide than the general population. The risk was increased after the diagnosis of mesothelioma, pancreatic cancer, esophageal cancer, and lung cancer [9]. This alarming observation was supported by a recent American study, which revealed that individuals diagnosed with cancer had a 4.44-fold greater risk of suicide than the general population [12]. The analysis indicated that the diagnosis of lung, head and neck, testis, and bladder cancers, as well as Hodgkin lymphoma, was associated with the highest suicide risk.

The existing literature indicates that the risk of suicide is higher among men and varies with age [4]. It also suggests that suicide risk is inversely associated with the time since cancer diagnosis, with the highest risk within the first six months, pinpointing when cancer care routes should give special attention to the patient’s psychological health needs [4,13]. However, most of the previously conducted analyses missed an opportunity to investigate the first six months post-diagnosis more closely, presenting novel findings. This may be partially explained by the insufficient power to describe risk in more fine-grained categories over time. A Swedish study investigated this and found the first six months to be a period of elevated risk of suicide in several cancers [14]. Nevertheless, this study was limited to eight cancer sites and focused only on the first year after diagnosis, reflecting the psychological stress induced by the diagnosis. The study did not present changes in suicide risk over time in the extended time frame, possibly missing the chance to assess the consequences of treatment or the burden of living with a progressive tumor. Newer studies are needed to probe high-risk periods in more fine-grained detail where possible.

In Central and Eastern European countries, identifying the target population and the best time for psycho-oncological intervention after a cancer diagnosis remains challenging. Previously published results cannot be extrapolated to patients from this region, who have distinct cultural factors that affect both the provision of services and attitudes toward patients with cancer (within services and within social networks) and thereby influence support provision. For instance, deeply ingrained stigma surrounding mental health in certain cultural contexts could discourage individuals from openly seeking psycho-oncological assistance. Additionally, traditional beliefs concerning cancer and fatalism might shape patients’ viewpoints on interventions. As a result, conducting a comprehensive overview specific to Central Europe becomes essential.

This study aimed to (1) determine whether Polish patients diagnosed with cancer have a greater risk of suicide than the general population; (2) identify subgroups at the highest risk; and (3) investigate time-based suicide risk post-diagnosis across detailed cancer site categories.

This is the first publication of the thoroughgoing Polish Suicidality in Cancer Patients (PolSCa) project. It should be regarded as a foundational study for multiple subsequent analyses of links between specific patient-related factors and well-defined cancer diseases to identify exposure-response patterns.

## 2. Materials and Methods

### 2.1. Source of the Data

Data on cancer cases (exposure) were obtained from the Polish National Cancer Registry (PLCR). The PLCR is a non-profit national institution responsible for statistical and epidemiological cancer research in Poland (population of 38.2 million in June 2021 [15]). The registry covers all the cancer cases diagnosed in Poland. Data are actively collected from hospitals, outpatient clinics, healthcare practitioners, and palliative care centers. Reporting of cancer cases to PLCR is mandatory. Physicians submit notifications that are later verified by qualified PLCR coders based on histopathology/cytological/cytometry examination results and finally passed through specific tools to validate the entries against the recommendations of the European Network of Cancer Registries. The PLCR system is based on a unique Polish personal identification number (PESEL) and avoids double coding for the same patient. The principles of PLCR operation are comprehensively described elsewhere [16].

The included cases encompassed all primary malignant neoplasms except non-melanoma skin cancers (C00-C43, C45-C76, and C80-C96 according to the ICD-10) diagnosed in patients ≥15 years old, grouped and labeled as in Table S1. Only the most recent diagnosis of a primary malignant neoplasm was included in the study of patients with two or more independent coexisting neoplasms. All cases diagnosed between 1 January 2009 and 31 December 2019 were included in the study. The follow-up endpoints were suicide (outcome), death due to other causes, or 31 December 2019, whichever occurred first.

Data on deaths due to suicide in the general Polish population, used to calculate the expected number of suicides, were obtained from the nationwide death registry (Statistics Poland, Warsaw, Poland) and encompassed all ICD-10 codes used for deaths due to suicide (X60–X84).

### 2.2. Statistical Analysis

The normality of continuous variables (age at diagnosis/suicide) was assessed using the Shapiro-Wilk test of normality. Descriptive statistics are presented as median and interquartile range (IQR) or mean and standard deviation (SD) for numeric variables and numbers and percentages for categorical variables.

The ratios of the observed to the expected number of deaths due to suicide, denoted as standardized mortality ratios (SMR), were calculated for all cancers overall and for specific cancer sites. Age-standardization was performed using person counts in five-year age groups (15–19, 20–24, …, and 75+) at the time of death due to suicide as weights. Age-period-sex-specific Polish national suicide incidence rates in the same five-year age groups were used as the reference. The 95% confidence intervals (CIs) were calculated assuming a Poisson distribution. To compute risks associated with age at the event, both for suicides in the general population and patients with cancer, individual-level data were split by calendar year, age, sex, and follow-up time into subject-sex-time intervals. Statistical analyses were performed using R (version 4.1.2).

### 2.3. Compliance with Ethical Standards

According to Polish legislation, individual-level data from the PLCR can be used for statistics in aggregate form and for scientific purposes. The PLCR obeys strict regulations to secure confidentiality and protect individuals. This study was conducted according to the Strengthening the Reporting of Observational Studies in Epidemiology (STROBE) guidelines [17].

## 3. Results

### 3.1. Patients’ Characteristics

The PolSCa study population included 1.43 million individuals (717 thousand men and 710 thousand women; Table S2) diagnosed with cancer between 1 January 2009 and 31 December 2019. In total, they contributed 3,627,793 person-years of observation. In this cohort, the most numerous groups of patients under observation were individuals diagnosed with tumors of the lung (210,584), breast (187,919), colorectum (178,267), prostate (142,079), and bladder (74,785). The least numerous groups included patients with cancer of the trachea (308), thymus (790), adrenal glands (1072), heart (1428), and appendicular skeleton (1472).

The overall mean age at the time of cancer diagnosis was 64.1 years (SD = 12.1; men 65.1 years, SD = 12.2; women 63.0 years, SD = 13.8; Table S2). The highest mean age at diagnosis was observed among patients with cancers of the vulva and vagina (70.4 years, SD = 12.4), and the lowest among patients with testicular cancer (34.6 years, SD = 11.7).

During follow-up, among all the patients diagnosed with primary cancer, there were 830 cases of suicide (683 men and 147 women). The highest number of suicide deaths was observed in patients with colorectal (134), prostate (133), lung (100), bladder (63), and head and neck (49) tumors. No suicides were reported among patients with adrenal gland, appendicular skeleton, thymus, or tracheal tumors.

Among men and women, the most common mechanisms of suicide were intentional self-harm by hanging, strangulation, and suffocation (85% of cases; Table S3). Less common means encompassed jumping from a high place (5%) and self-poisoning and exposure to alcohol (3%).

### 3.2. Overall Findings

The risk of suicide among patients diagnosed with cancer was significantly higher than that among the general population (SMR = 1.34, 95% CI 1.25 to 1.43, Table 1). Among women, this difference was higher (SMR = 1.49, 95% CI 1.26 to 1.75) than among men (SMR = 1.31, 95% CI 1.21 to 1.41).

### 3.3. Risk of Suicide by Age Group

The risk of suicide in both sexes was higher among older patients. Patients diagnosed aged 75–99 years were at the highest risk (SMR = 1.64, 95% CI = 1.43 to 1.87, Table 1). Among men, a significantly higher risk was observed in the 65–74 and 75–99 age groups (SMR = 1.47, 95% CI 1.29 to 1.68, and SMR = 1.70, 95% CI 1.47 to 1.95, respectively). In contrast, among women, the highest risk was observed in the 55–64 and 65–74 age groups (SMR = 1.43, 95% CI 1.06 to 1.88, and SMR = 1.75, 95% CI 1.26 to 2.38, respectively).

### 3.4. Risk of Suicide by Time since Cancer Diagnosis

The highest risk of suicide was observed in the first six months after diagnosis (SMR = 1.94, 95% CI 1.69 to 2.21, Table 1). Within this period, the standardized risk of suicide death among women was 2.33 (95% CI 1.63 to 3.22) and among men was 1.88 (95% CI 1.61 to 2.17). Overall, the risk of suicide was elevated for two years after diagnosis and decreased with time. After 5–10 years post-diagnosis, the risk of suicide among men increased, which was also reflected in the increase in the overall risk.

### 3.5. Risk of Suicide by the Site of the Last Primary Cancer

The age- and sex-standardized risk of suicide was significantly increased among patients with esophagus (SMR = 2.94, 95% CI 1.47 to 5.26), stomach (SMR = 2.70, 95% CI 2.00 to 3.57), cervix uteri (SMR = 2.20, 95% CI 1.06 to 4.05), head and neck (SMR = 2.06, 1.52 to 2.72), lung (SMR = 1.59, 95% CI 1.29 to 1.93), colorectum (SMR = 1.46, 95% CI 1.22 to 1.73), and breast tumors (SMR = 1.35, 95% CI 1.00 to 1.79) compared to the general population (Table 2). None of the cancer diagnoses was associated with a significantly lower risk of suicide than that of the general population.

### 3.6. Risk of Suicide by Sex

The risk of death by suicide varied according to patients’ sex. Among men, a significantly increased risk was observed for cancers of the esophagus (SMR = 2.80, 95% CI 1.34 to 5.16), stomach (SMR = 2.77, 95% CI 2.03 to 3.70), head and neck (SMR = 2.11, 95% CI 1.55 to 2.81), lung (SMR = 1.54, 95% CI 1.24 to 1.89), and colorectum (SMR = 1.49, 95% CI 1.23 to 1.78) (Table 2). Whereas, among women, the risk was increased for tumors of the larynx (SMR = 7.97, 95% CI 2.17 to 20.41), lymphoid tissue (SMR = 3.37, 95% CI 1.54 to 6.40), cervix uteri (SMR = 2.20, 95% CI 1.06 to 4.05), lung (SMR = 2.14, 95% CI 1.07 to 3.84), and breast (SMR = 1.37, 95% CI 1.01 to 1.82). Notably, the only cancer in which both men and women had a significantly increased risk of suicide was lung cancer, with a higher risk among women.

### 3.7. Risk of Suicide by the Site of the Last Primary Cancer and by Time since Cancer Diagnosis

Within the first six months after diagnosis, an increased risk of suicide was observed for the heart and pleura (SMR = 19.15, 95% CI 2.32 to 69.18), unspecified site (SMR = 3.99, 95% CI 1.09 to 10.22), esophagus (SMR = 3.34, 95% CI 1.08 to 7.79), head and neck (SMR = 3.02, 95% CI 1.69 to 4.99), gastric (SMR = 2.88, 95% CI 1.57 to 4.83), lymphoma (SMR = 2.61, 95% CI 1.12 to 5.13), laryngeal (SMR = 2.38, 95% CI 1.03 to 4.68), pulmonary (SMR = 2.37, 95% CI 1.74 to 3.16), and colorectal cancers (SMR = 1.84, 95% CI 1.23 to 2.64) (Table 3). Six months to one year after diagnosis, an increased risk was observed in patients with cancers of the ovary (SMR = 5.63, 95% CI 1.54 to 14.43), stomach (SMR = 3.87, 95% CI 2.00 to 6.76), head and neck (SMR = 2.52, 95% CI 1.15 to 4.78), and breast (SMR = 2.41, 95% CI 1.16 to 4.43). In the period from the second to the third year after diagnosis, an increased risk was observed in patients with colorectal tumors (SMR = 1.86, 95% CI 1.21 to 2.72). In the third to fifth years after diagnosis, the risk increased in patients with gastric cancer (SMR = 2.78, 95% CI 1.12 to 5.73). From the fifth to tenth year post-diagnosis, an increased risk was observed in patients with pancreatic cancer (SMR = 8.55, 95% CI 1.76 to 24.99) and head and neck cancer (SMR = 3.04, 95% CI 1.39 to 5.77).

## 4. Discussion

### 4.1. Added Value of This Study

To our knowledge, this is the most thorough examination of lethal suicide behaviors after cancer diagnosis in Central Europe, with estimates for over 1.4 million adult patients with cancer. It improves upon earlier research in this area in terms of scale (study population), method (SIR-type epidemiological model that uses age-period-sex-specific national suicide incidence rates as a reference), and generalizability (since it deploys the entire national population).

### 4.2. Main Research Findings in the Context of the Literature

The results of this study indicate that the overall risk of suicide among patients with cancer is 34% higher than that of the general population. These results seem consistent with other European studies, which found an increase in suicide risk of 20% in England, 23% in Austria, and 55% in Norway [9,11,18]. However, the risk observed in this investigation was far below that reported in the USA and Korea (106% and 100%, respectively) [8,10]. Since the deployed SMR calculation methods are relatively comparable, one might hypothesize that the discrepancies between European and non-European observations could be attributed to two possible causes. The first is the difference in healthcare systems. While most European and South Korean systems are universal and funded mainly through government subsidies, the United States has no universal healthcare coverage, and cancer diagnosis is associated with the financial stress of patients and their relatives [19,20]. Another possible explanation is the potential cultural differences that influence factors related to hope and cancer stigma. In England, where patients with cancer have a significantly lower risk of suicide than in Korea, cancer stigma is correspondingly lower [21,22]. Due to the lack of data, reference to cancer stigma among Polish patients with cancer is impossible.

As anticipated, suicide risk varied according to gender. However, contrary to expectations, this study found a higher overall SMR among women than among men. This is surprising because, in the general Polish population, the risk of suicide is higher in men [23]. Such a phenomenon has not been previously described and is contrary to previous studies, which have suggested that men with cancer are characterized by a higher risk of suicide [8,9]. It is difficult to explain this result, but it might be related to variations in cancer stigma, cancer economic burden, differing experiences, impacts of disfigurement, role loss, and functional impairment by sex. These findings need to be scrutinized.

Another interesting finding was that, compared to other age groups, the highest standardized risk of suicide was observed among the oldest patients (age group 75–99). In the literature, the age group with the highest risk of suicide after cancer diagnosis has been subject to considerable variation (Estonian men 15–49, American men, and women 85+ years) [8,24]. The reason for the highest risk among Polish seniors is unclear. It may be linked to ageism in the health care system, reflected in age-based bias in cancer screening and treatment, leading to discrepancies in care that are not justified by clinical evidence or best practice. There is also evidence that the overall risk of attempted suicide in late life is associated with sex, depression, other psychiatric conditions, marital status, living status, education, drug/alcohol abuse, social isolation, and economic status [25]. Additional studies are needed to evaluate the possible associations with these risk factors and develop a full picture of suicidality in elderly patients with cancer.

This study offers important insights into variations in suicide risk over time since diagnosis. As in previous studies [9,11], the highest risk of suicide was observed in the first six months after a cancer diagnosis. However, one of the more significant findings of this study is that the risk of suicide by time after cancer diagnosis differs depending on the cancer site categories. Within the first half a year after diagnosis, most likely reflecting psychological stress induced by the diagnosis, the risk was higher for cancers of the heart and pleura, unspecified site, esophagus, head and neck, stomach, lymphoma, larynx, lung, and colorectum. Later categories (after the first six months post-diagnosis), possibly reflecting waiting for treatment, consequences of therapy, the burden of living with progressive tumors, disfigurement, insufficient pain management, and the economic burden of cancer, encompassed ovarian, gastric, head and neck, breast, colorectal, and pancreatic cancers. Further investigation of these variations is required.

Finally, the risk of suicide varied according to the cancer site. It was higher in men with cancers of the esophagus, stomach, head and neck, lung, and colorectum and in women with tumors of the larynx, lymphoid tissue, cervix uteri, lung, and breast. Possible explanations for these results may be low predicted survival (lung [26]), physical disfigurement (head and neck), low quality of life after treatment (larynx and esophagus), chronic pain, and barriers to accessing palliative care. Comparing opioid consumption in Poland with other Western European countries provides context for access to palliative care in Poland. Total opioid consumption in Poland, described as the Defined Daily Dose, defined as the assumed average maintenance dose per day for a drug used for its main indication in adults, was 1357 and 1840 daily doses in 2004–2006 and 2014–2016, respectively [27]. In comparison, the numbers were considerably higher in other countries during 2014–2016, with the UK at 8214, the EU overall at 8967, the Netherlands at 12,198, and Germany at 21,346 daily doses. Therefore, further studies with a greater focus on socioeconomic variables and palliative care access are suggested

### 4.3. The Current Situation in Mental Care in Poland

In Poland, one does not need a referral letter to consult a psychiatrist. However, to make an appointment with a psychologist or psychotherapist, a referral is required. Although there is no health benefit basket in force in the country, including mental health care, it should be noted that Poland has one of the lowest rates of access to a psychiatrist in Europe, with only nine doctors per 100,000 inhabitants [28]. For comparison, in Nordic countries, Germany, and Benelux countries, this indicator is twice as high. Additionally, from 2010 to 2021, spending on mental health accounted for less than four percent of the Polish National Health Fund’s budget [29]. In Germany and France, governmental expenditures on mental health as a percentage of the total government expenditure on health are 11% and 13%, respectively [30].

There are no national-level strategies to address suicide prevention or provide mental health support to Polish patients with cancer. Oncology hospitals have psychiatric facilities but are usually understaffed and underfinanced. There is also a lack of specialist and systemic training for medical personnel (nurses and patient assistants) aimed at the early detection of a possible increased risk of suicide among patients. There are training opportunities in this field, but they usually involve expenses that must be covered out of the trainees’ own pockets.

Polish non-governmental organizations aim to help people in a suicide crisis, as well as those who experience mental difficulties or mourn the suicidal death of a loved one. Most of these organizations target the general population free of charge and anonymously. There are also organizations for oncology patients offering free one-time or regular sessions with a psycho-oncologist, consultations for families and friends of cancer patients, workshops for people after cancer treatment, and psychological help for people grieving after the loss of a loved one. However, it should be noted that finding information about these institutions requires Internet access. For various reasons, some patients do not have access to the computer/Internet, cannot search for information on the Internet, or do not know that they can look for it there. In such cases, they rely on help from the public healthcare system.

### 4.4. Implications for Clinical Practice

Prior to this study, predicting groups of increased suicide risk among Polish patients with cancer was challenging. The PolSCa study deepened the understanding of individuals facing these challenges.

Our findings suggest that women and seniors require focused psycho-oncological care, while highlighting the critical importance of interventions within the first six months post-diagnosis. This critical window of intervention calls for resource allocation that supports timely and accessible mental health services for women and seniors diagnosed with cancer. Implementing comprehensive psycho-oncological support programs during this period can significantly improve mental well-being and contribute to holistic cancer care.

Moreover, our results emphasize the significance of providing comprehensive psychiatric/psychological support, recognizing the complexities of individual experiences, including instances (cancer sites) where individuals may be considering end-of-life decisions. Recognizing the intersection of mental health and end-of-life considerations is pivotal for patient-centered care. Mental health professionals can collaborate with policymakers to develop guidelines that address the unique psychological needs of patients facing life-limiting diagnoses, ensuring compassionate support and informed decision-making.

### 4.5. Implications for Policy Makers and Other Stakeholders

Many cases of suicide are potentially preventable by tackling modifiable risk factors. Established preventive strategies should be one of the main components of the treatment standards for these patients. This cannot be overlooked because it is essential to minimize the suicide incidence rate among patients with cancer. However, patients with cancer are a particularly vulnerable population, and knowledge of suicidality in other groups cannot be extrapolated to this population. Suicide prevention in this group might require new approaches based on the suicide risk factors in these patients.

In some countries, clinical practice guidelines recommend suicide risk screening in patients with cancer by applying a particular depression rating scale. The American Society of Clinical Oncology suggests screening for depressive symptoms regularly [31]. In addition, Canadian clinical practice guidelines for managing depression in adult cancer patients assess suicidal intent by applying depression rating scales [32]. The standard of care in many settings is tiered, starting with training cancer clinical nurses to identify suicidal distress, for example, by the screening methods mentioned above. The next tier would be to see a counselor or psychologist with psychiatric input only for the most severe (steroid-induced mania, psychotic depression, severe anxiety precluding cancer treatment, and intractable depression). However, it is unclear whether these recommended interventions effectively reduce suicidality among cancer patients. According to a systematic review of interventions to prevent suicidal behaviors and ideation in patients with cancer, there is little evidence to confirm the effects of suicide prevention strategies [13]. There is a need to explore new treatment strategies that focus on the unique suicide risk factors among patients with cancer.

In Poland, adequate measures must be implemented at a national level. Therefore, it is necessary to create a national strategy for mental health care in oncology. Suicide risk-screening tools should also be developed. It should also be emphasized that in cases of increased suicide risk, evidence-based mental health interventions should be triggered to prevent this outcome. Moreover, in addition to policymakers, non-governmental organizations that have helped patients with cancer may also play a role in improving the situation. They should target their psycho-oncological help at digitally excluded patients, to whom assistance can be provided traditionally through printed or multimedia materials available in health care centers or conventional media.

Reducing premature mortality from non-communicable diseases, including cancer, by one-third by 2030 is one of the goals of the United Nations’ 2030 Agenda for Sustainable Development. One of the steps to fulfill this aim is the collaboration of epidemiologists and healthcare providers in identifying risk factors associated with suicidal behaviors among patients with cancer and providing targeted psycho-oncological support to the most vulnerable individuals. There is room for further progress in determining the possible causes of increased suicide in Polish patients with cancer.

Future research endeavors could delve into the interplay between cancer treatment modalities, psychological distress, and suicide risk. Exploring how treatment-related factors influence patients’ mental health can guide the development of personalized interventions aimed at reducing suicide risk. Identifying the causes and subsequent efforts to minimize risk should eventually minimize suicide-related social and familial consequences. Further investigations could shed light on subgroups at heightened risk, such as patients with specific cancer types or demographic characteristics. Tailoring support strategies for these high-risk groups can not only mitigate individual suffering but also alleviate the broader societal impact of suicide-related consequences.

The findings of this study comprehensively support responsive service provision in Poland. Additionally, the study’s outcomes hold significant implications for Central Europe’s public health and psychosocial oncology. By identifying high-risk subgroups and emphasizing cross-border applicability, policymakers can shape suicide prevention strategies. These insights foster collaborative efforts, optimizing mental health support and resource allocation in alignment with regional health agendas.

### 4.6. Strengths and Limitations of the Study

The potential source of bias in this study was the loss to follow-up. However, since the PLCR is a population-based registry deploying unique personal identification numbers for registration and conducting yearly follow-ups using the Polish Population Information System, loss of follow-up in a strict sense is not anticipated. Still, there remains an unknown number of unregistered deaths of individuals emigrating permanently from Poland with no further legal connection with the country. Nevertheless, for such cases, a healthy migrant effect most likely applies, and there are no grounds to assume that even if previously diagnosed with cancer, these patients’ suicide risk would differ from that of other patients diagnosed with the same disease.

The two main potential confounders in this study were sex and age; hence, we performed sex- and age-standardization when calculating the overall risks and age-standardization when calculating risk by sex. In addition, the timing of suicides related to terminal cancers poses potential confounding due to treatment differences. Other potential confounding factors included a history of previous suicide attempts, a history of substance abuse, a history of mental health conditions, relationship problems, a history of legal or disciplinary problems, a history of the recent death of a family member or a close friend, or a history of a disability other than cancer. Owing to a lack of data on these factors, we could not consider them during the analysis, which could be a limitation of the study.

The significant advantage of the presented research is both the good quality of the data on exposure (precision of registration of cancer cases in the PLCR and verification by trained registrars) and the outcome (accuracy of causes of death certification by the Polish Population Information System). To the authors’ knowledge, the PolSCa study is the first Polish study to assess the relationship between cancer diagnosis and the risk of suicide in the entire national population, allowing for the generalizability of the results. The study population constitutes a significant sample that is fully covered by universal access to healthcare, preventing socioeconomic biases. In addition, the cohort design of the study, data linkage based on unique personal identity codes, an extended period of follow-up, and low loss to follow-up should be regarded as the strengths of the presented research.

## 5. Conclusions

Patients diagnosed with cancer have a significantly higher risk of suicide than those in the general population. The risk is higher among women than men and highest in individuals aged 75–99. The risk of suicide is inversely associated with the time since cancer diagnosis and is greatest within the first six months after diagnosis. Among men, it is the highest in cases of cancers of the esophagus, stomach, head and neck, lung, and colorectum, and among women, in tumors of the larynx, lymphoid tissue, cervix uteri, lung, and breast.

Our comprehensive analyses of the impact of cancer diagnosis on suicide risk are critical for public health expenditure decisions in Central Europe. Furthermore, identifying specific cancer sites and cancer-sex combinations that contribute the most to the burden (according to our estimations) may explicitly advise policy targets and planning, focusing on expanding suicide prevention and informing research priorities in the field of psychosocial oncology.

## Figures and Tables

**Table 1:** Deaths due to suicide among patients with cancer—standardized mortality ratios (SMR) and 95% confidence intervals (95% CI) by sex, age group, and time since diagnosis.

	Overall			Men			Women		
	Observed	Expected	SMR (95% CI)	Observed	Expected	SMR (95% CI)	Observed	Expected	SMR (95% CI)
All cancers *	830	620	1.34 (1.25–1.43)	683	521	1.31 (1.21–1.41)	147	99	1.49 (1.26–1.75)
**Age at suicide group [years]**									
[15, 45)	45	36	1.24 (0.91–1.66)	36	30	1.18 (0.83–1.64)	9	6	1.57 (0.72–2.98)
[45, 55)	86	67	1.28 (1.03–1.58)	63	51	1.24 (0.95–1.59)	23	16	1.42 (0.90–2.12)
[55, 65)	210	205	1.03 (0.89–1.17)	159	169	0.94 (0.80–1.10)	51	36	1.43 (1.06–1.88)
[65, 75)	271	179	1.51 (1.34–1.70)	230	156	1.47 (1.29–1.68)	41	23	1.75 (1.26–2.38)
[75, 99]	218	133	1.64 (1.43–1.87)	195	115	1.70 (1.47–1.95)	23	18	1.28 (0.81–1.93)
**Time since diagnosis [years]**									
[0.0, 0.5)	220	114	1.94 (1.69–2.21)	184	98	1.88 (1.61–2.17)	36	16	2.33 (1.63–3.22)
[0.5, 1.0)	122	87	1.41 (1.17–1.68)	94	74	1.27 (1.03–1.56)	28	13	2.23 (1.48–3.22)
[1.0, 2.0)	154	127	1.21 (1.03–1.42)	127	108	1.18 (0.98–1.40)	27	20	1.37 (0.90–1.99)
[2.0, 3.0)	91	91	1.00 (0.81–1.23)	74	76	0.97 (0.76–1.22)	17	15	1.13 (0.66–1.81)
[3.0, 5.0)	135	116	1.16 (0.97–1.37)	112	96	1.17 (0.96–1.40)	23	20	1.13 (0.72–1.70)
[5.0, 10.0]	105	86	1.23 (1.00–1.49)	90	70	1.29 (1.04–1.59)	15	16	0.95 (0.53–1.56)

* All primary malignant neoplasms except non-melanoma skin cancers (C00–C43, C45–C76, and C80–C96 according to the ICD-10).

**Table 2:** Deaths due to suicide among patients with cancer—standardized mortality ratios (SMR) and 95% confidence intervals (95% CI) by cancer site and sex.

ICD	Site	Overall			Men			Women		
		Observed	Expected	SMR (95% CI)	Observed	Expected	SMR (95% CI)	Observed	Expected	SMR (95% CI)
C00–C14 + C30	Head and neck	49	24	2.06 (1.52–2.72)	47	22	2.11 (1.55–2.81)	2	2	1.26 (0.15–4.55)
C15	Esophagus	11	4	2.94 (1.47–5.26)	10	4	2.80 (1.34–5.16)	1	0	5.75 (0.15–32.03)
C16	Stomach	49	18	2.70 (2.00–3.57)	46	17	2.77 (2.03–3.70)	3	2	1.90 (0.39–5.56)
C17	Small intestine	2	2	1.30 (0.16–4.70)	2	1	1.49 (0.18–5.37)	0	0	0.00 (0.00–19.18)
C18–C20	Colorectum	134	92	1.46 (1.22–1.73)	121	81	1.49 (1.23–1.78)	13	10	1.25 (0.66–2.13)
C21	Anus	2	1	2.09 (0.25–7.56)	1	1	1.46 (0.04–8.15)	1	0	3.69 (0.09–20.55)
C22–24	Liver and gallbladder	8	6	1.37 (0.59–2.71)	7	5	1.43 (0.57–2.94)	1	1	1.08 (0.03–6.04)
C25	Pancreas	10	6	1.77 (0.85–3.25)	9	5	1.84 (0.84–3.50)	1	1	1.29 (0.03–7.20)
C32	Larynx	28	20	1.42 (0.95–2.06)	24	19	1.25 (0.80–1.86)	4	1	7.97 (2.17–20.41)
C33	Trachea	0	0	0.00 (0.00–38.19)	0	0	0.00 (0.00–42.95)	0	0	0.00 (0.00–344.39)
C34	Lung	100	63	1.59 (1.29–1.93)	89	58	1.54 (1.24–1.89)	11	5	2.14 (1.07–3.84)
C37	Thymus	0	0	0.00 (0.00–8.95)	0	0	0.00 (0.00–10.47)	0	0	0.00 (0.00–61.62)
C38	Heart and pleura	2	0	4.34 (0.53–15.69)	2	0	4.83 (0.59–17.45)	0	0	0.00 (0.00–79.18)
C40	Bone—limbs	0	1	0.00 (0.00–4.55)	0	1	0.00 (0.00–5.12)	0	0	0.00 (0.00–40.91)
C41	Bone—axial skeleton	1	1	1.32 (0.03–7.35)	1	1	1.52 (0.04–8.50)	0	0	0.00 (0.00–36.05)
C43	Melanoma	18	18	1.02 (0.60–1.61)	17	15	1.14 (0.66–1.83)	1	3	0.36 (0.01–2.01)
C45–C49	Soft tissues	6	6	1.06 (0.39–2.31)	6	5	1.21 (0.44–2.64)	0	1	0.00 (0.00–5.19)
C50	Breast	48	36	1.35 (1.00–1.79)	1	1	0.76 (0.02–4.22)	47	34	1.37 (1.01–1.82)
C51–C52	Vulva and vagina	3	1	3.95 (0.82–11.55)	-	-	(-)	3	1	3.95 (0.82–11.55)
C53	Cervix uteri	10	5	2.20 (1.06–4.05)	-	-	(-)	10	5	2.20 (1.06–4.05)
C54–C55	Corpus uteri	12	11	1.08 (0.56–1.89)	-	-	(-)	12	11	1.08 (0.56–1.89)
C56	Ovary	8	5	1.53 (0.66–3.02)	-	-	(-)	8	5	1.53 (0.66–3.02)
C60	Penis	2	2	0.87 (0.11–3.15)	2	2	0.87 (0.11–3.15)	-	-	(-)
C61	Prostate	133	133	1.00 (0.83–1.18)	133	133	1.00 (0.83–1.18)	-	-	(-)
C62	Testis	18	14	1.32 (0.78–2.09)	18	14	1.32 (0.78–2.09)	-	-	(-)
C64	Kidney	32	33	0.96 (0.66–1.36)	27	30	0.90 (0.59–1.31)	5	3	1.53 (0.50–3.57)
C65–C67	Bladder	63	53	1.20 (0.92–1.53)	61	50	1.22 (0.93–1.57)	2	3	0.77 (0.09–2.78)
C69–C72	Central nervous system	11	11	0.96 (0.48–1.72)	9	10	0.91 (0.42–1.73)	2	2	1.27 (0.15–4.57)
C73	Thyroid	8	11	0.76 (0.33–1.49)	2	6	0.33 (0.04–1.19)	6	5	1.32 (0.49–2.88)
C74	Adrenal gland	0	0	0.00 (0.00–7.97)	0	0	0.00 (0.00–9.84)	0	0	0.00 (0.00–41.97)
C76, C80	Unspecified site	10	6	1.77 (0.85–3.25)	8	5	1.63 (0.71–3.22)	2	1	2.60 (0.32–9.40)
C81–C88	Lymphoma	23	21	1.11 (0.70–1.67)	14	18	0.78 (0.42–1.30)	9	3	3.37 (1.54–6.40)
C90	Multiple myeloma	11	7	1.67 (0.84–2.99)	9	6	1.62 (0.74–3.07)	2	1	2.00 (0.24–7.22)
C91–C96	Leukemia	18	17	1.07 (0.63–1.68)	17	15	1.13 (0.66–1.81)	1	2	0.54 (0.01–3.01)

**Table 3:** Deaths due to suicide among patients with cancer—standardized mortality ratios (SMR) and 95% confidence intervals (95% CI) by cancer site and time since diagnosis.

ICD	Site	Time Since Diagnosis [Years]	Observed	Expected	SMR (95% CI)
	All cancers *	[0.0, 0.5)	220	114	1.94 (1.69–2.21)
		[0.5, 1.0)	122	87	1.41 (1.17–1.68)
		[1.0, 2.0)	154	127	1.21 (1.03–1.42)
		[2.0, 3.0)	91	91	1.00 (0.81–1.23)
		[3.0, 5.0)	135	116	1.16 (0.97–1.37)
		[5.0, 10.0]	105	86	1.23 (1.00–1.49)
C00–C14 + C30	Head and neck	[0.0, 0.5)	15	5	3.02 (1.69–4.99)
		[0.5, 1.0)	9	4	2.52 (1.15–4.78)
		[1.0, 2.0)	7	5	1.45 (0.58–2.98)
		[2.0, 3.0)	2	3	0.60 (0.07–2.18)
		[3.0, 5.0)	7	4	1.68 (0.68–3.46)
		[5.0, 10.0]	9	3	3.04 (1.39–5.77)
C15	Esophagus	[0.0, 0.5)	5	2	3.34 (1.08–7.79)
		[0.5, 1.0)	3	1	4.01 (0.83–11.72)
		[1.0, 2.0)	3	1	4.52 (0.93–13.2)
		[2.0, 3.0)	0	0	0.00 (0.00–11.83)
		[3.0, 5.0)	0	0	0.00 (0.00–11.68)
		[5.0, 10.0]	0	0	0.00 (0.00–18.19)
C16	Stomach	[0.0, 0.5)	14	5	2.88 (1.57–4.83)
		[0.5, 1.0)	12	3	3.87 (2.00–6.76)
		[1.0, 2.0)	7	4	1.87 (0.75–3.85)
		[2.0, 3.0)	4	2	1.82 (0.49–4.65)
		[3.0, 5.0)	7	3	2.78 (1.12–5.73)
		[5.0, 10.0]	5	2	2.87 (0.93–6.69)
C17	Small intestine	[0.0, 0.5)	1	0	3.81 (0.10–21.25)
		[0.5, 1.0)	0	0	0.00 (0.00–17.73)
		[1.0, 2.0)	0	0	0.00 (0.00–11.45)
		[2.0, 3.0)	1	0	4.28 (0.11–23.87)
		[3.0, 5.0)	0	0	0.00 (0.00–12.27)
		[5.0,10.0]	0	0	0.00 (0.00–17.42)
C18–C20	Colorectum	[0.0, 0.5)	29	16	1.84 (1.23–2.64)
		[0.5, 1.0)	15	13	1.17 (0.65–1.93)
		[1.0, 2.0)	27	20	1.37 (0.90–1.99)
		[2.0, 3.0)	26	14	1.86 (1.21–2.72)
		[3.0, 5.0)	20	17	1.16 (0.71–1.79)
		[5.0, 10.0]	17	12	1.39 (0.81–2.23)
C21	Anus	[0.0, 0.5)	1	0	5.78 (0.15–32.2)
		[0.5, 1.0)	0	0	0.00 (0.00–26.89)
		[1.0, 2.0)	0	0	0.00 (0.00–18.39)
		[2.0, 3.0)	0	0	0.00 (0.00–26.44)
		[3.0, 5.0)	1	0	5.68 (0.14–31.66)
		[5.0, 10.0]	0	0	0.00 (0.00–28.66)
C22–C24	Liver and gallbladder	[0.0, 0.5)	2	2	1.10 (0.13–3.96)
		[0.5, 1.0)	1	1	0.96 (0.02–5.37)
		[1.0, 2.0)	3	1	2.54 (0.52–7.43)
		[2.0, 3.0)	2	1	3.06 (0.37–11.05)
		[3.0, 5.0)	0	1	0.00 (0.00–5.32)
		[5.0, 10.0]	0	0	0.00 (0.00–8.39)
C25	Pancreas	[0.0, 0.5)	6	2	2.66 (0.98–5.8)
		[0.5, 1.0)	0	1	0.00 (0.00–3.41)
		[1.0, 2.0)	1	1	1.02 (0.03–5.67)
		[2.0, 3.0)	0	0	0.00 (0.00–7.69)
		[3.0, 5.0)	0	1	0.00 (0.00–7.29)
		[5.0, 10.0]	3	0	8.55 (1.76–24.99)
C32	Larynx	[0.0, 0.5)	8	3	2.38 (1.03–4.68)
		[0.5, 1.0)	6	3	2.20 (0.81–4.79)
		[1.0, 2.0)	8	4	1.97 (0.85–3.87)
		[2.0, 3.0)	1	3	0.34 (0.01–1.89)
		[3.0, 5.0)	4	4	1.06 (0.29–2.71)
		[5.0, 10.0]	1	3	0.36 (0.01–1.99)
C34	Lung	[0.0, 0.5)	47	20	2.37 (1.74–3.16)
		[0.5, 1.0)	18	12	1.54 (0.91–2.43)
		[1.0, 2.0)	12	13	0.96 (0.49–1.67)
		[2.0, 3.0)	7	7	1.03 (0.41–2.12)
		[3.0, 5.0)	9	7	1.23 (0.56–2.33)
		[5.0, 10.0]	6	5	1.25 (0.46–2.73)
C38	Heart and pleura	[0.0, 0.5)	2	0	19.15 (2.32–69.18)
		[0.5, 1.0)	0	0	0.00 (0.00–60.35)
		[1.0, 2.0)	0	0	0.00 (0.00–43.58)
		[2.0, 3.0)	0	0	0.00 (0.00–61.5)
		[3.0, 5.0)	0	0	0.00 (0.00–45.34)
		[5.0, 10.0]	0	0	0.00 (0.00–53.45)
C41	Bone–axial skeleton	[0.0, 0.5)	0	0	0.00 (0.00–27.59)
		[0.5, 1.0)	0	0	0.00 (0.00–35.98)
		[1.0, 2.0)	1	0	6.49 (0.16–36.15)
		[2.0, 3.0)	0	0	0.00 (0.00–32.89)
		[3.0, 5.0)	0	0	0.00 (0.00–25.27)
		[5.0,10.0]	0	0	0.00 (0.00–33.63)
C43	Melanoma	[0.0, 0.5)	4	3	1.47 (0.40–3.76)
		[0.5, 1.0)	2	2	0.85 (0.10–3.08)
		[1.0, 2.0)	1	4	0.27 (0.01–1.49)
		[2.0, 3.0)	2	3	0.73 (0.09–2.62)
		[3.0, 5.0)	5	4	1.42 (0.46–3.32)
		[5.0, 10.0]	3	3	1.16 (0.24–3.40)
C45–C49	Soft tissues	[0.0, 0.5)	1	1	0.91 (0.02–5.08)
		[0.5, 1.0)	1	1	1.19 (0.03–6.62)
		[1.0, 2.0)	0	1	0.00 (0.00–3.08)
		[2.0, 3.0)	2	1	2.46 (0.30–8.88)
		[3.0, 5.0)	0	1	0.00 (0.00–3.76)
		[5.0, 10.0]	2	1	2.72 (0.33–9.83)
C50	Breast	[0.0, 0.5)	9	5	1.95 (0.89–3.71)
		[0.5, 1.0)	10	4	2.41 (1.16–4.43)
		[1.0, 2.0)	10	7	1.42 (0.68–2.61)
		[2.0, 3.0)	8	6	1.41 (0.61–2.79)
		[3.0, 5.0)	9	8	1.14 (0.52–2.17)
		[5.0, 10.0]	2	6	0.32 (0.04–1.16)
C51–C52	Vulva and vagina	[0.0, 0.5)	0	0	0.00 (0.00–26.57)
		[0.5, 1.0)	1	0	9.29 (0.24–51.75)
		[1.0, 2.0)	0	0	0.00 (0.00–23.95)
		[2.0, 3.0)	1	0	9.06 (0.23–50.47)
		[3.0, 5.0)	0	0	0.00 (0.00–25.72)
		[5.0, 10.0]	1	0	9.56 (0.24–53.27)
C53	Cervix uteri	[0.0, 0.5)	2	1	2.94 (0.36–10.63)
		[0.5, 1.0)	1	1	1.76 (0.04–9.78)
		[1.0, 2.0)	2	1	2.25 (0.27–8.13)
		[2.0, 3.0)	1	1	1.48 (0.04–8.27)
		[3.0, 5.0)	1	1	1.06 (0.03–5.92)
		[5.0, 10.0]	3	1	3.80 (0.78–11.12)
C54	Corpus uteri	[0.0, 0.5)	4	1	2.71 (0.74–6.93)
		[0.5, 1.0)	1	1	0.76 (0.02–4.25)
		[1.0, 2.0)	3	2	1.37 (0.28–4.00)
		[2.0, 3.0)	0	2	0.00 (0.00–2.11)
		[3.0, 5.0)	3	2	1.24 (0.26–3.62)
		[5.0, 10.0]	0	2	0.00 (0.00–1.91)
C56	Ovary	[0.0, 0.5)	2	1	2.33 (0.28–8.43)
		[0.5, 1.0)	4	1	5.63 (1.54–14.43)
		[1.0, 2.0)	0	1	0.00 (0.00–3.32)
		[2.0, 3.0)	1	1	1.24 (0.03–6.9)
		[3.0, 5.0)	0	1	0.00 (0.00–3.66)
		[5.0, 10.0]	1	1	1.39 (0.04–7.74)
C60	Penis	[0.0, 0.5)	0	0	0.00 (0.00–9.67)
		[0.5, 1.0)	1	0	3.20 (0.08–17.85)
		[1.0, 2.0)	0	0	0.00 (0.00–7.83)
		[2.0, 3.0)	0	0	0.00 (0.00–10.8)
		[3.0, 5.0)	1	0	2.23 (0.06–12.42)
		[5.0, 10.0]	0	0	0.00 (0.00–10.81)
C61	Prostate	[0.0, 0.5)	25	20	1.27 (0.82–1.87)
		[0.5, 1.0)	15	17	0.87 (0.48–1.43)
		[1.0, 2.0)	38	28	1.34 (0.95–1.85)
		[2.0, 3.0)	11	21	0.52 (0.26–0.93)
		[3.0, 5.0)	28	27	1.03 (0.68–1.48)
		[5.0, 10.0]	16	20	0.82 (0.47–1.33)
C62	Testis	[0.0, 0.5)	2	1	1.38 (0.17–4.97)
		[0.5, 1.0)	2	1	1.48 (0.18–5.36)
		[1.0, 2.0)	1	2	0.41 (0.01–2.30)
		[2.0, 3.0)	4	2	1.92 (0.52–4.93)
		[3.0, 5.0)	3	3	0.93 (0.19–2.73)
		[5.0, 10.0]	6	3	1.95 (0.71–4.23)
C64	Kidney	[0.0, 0.5)	4	5	0.79 (0.22–2.03)
		[0.5, 1.0)	5	4	1.20 (0.39–2.80)
		[1.0, 2.0)	7	7	1.04 (0.42–2.15)
		[2.0, 3.0)	3	5	0.58 (0.12–1.70)
		[3.0, 5.0)	7	7	1.02 (0.41–2.10)
		[5.0, 10.0]	6	5	1.15 (0.42–2.50)
C65–C67	Bladder	[0.0, 0.5)	15	8	1.77 (0.99–2.92)
		[0.5, 1.0)	4	7	0.58 (0.16–1.48)
		[1.0, 2.0)	12	11	1.11 (0.57–1.94)
		[2.0, 3.0)	6	8	0.75 (0.27–1.63)
		[3.0, 5.0)	16	11	1.52 (0.87–2.47)
		[5.0, 10.0]	10	8	1.29 (0.62–2.37)
C69–C72	Central nervous system	[0.0, 0.5)	6	3	2.38 (0.87–5.18)
		[0.5, 1.0)	2	2	1.18 (0.14–4.28)
		[1.0, 2.0)	1	2	0.45 (0.01–2.52)
		[2.0, 3.0)	0	2	0.00 (0.00–2.44)
		[3.0, 5.0)	2	2	1.03 (0.12–3.71)
		[5.0, 10.0]	0	2	0.00 (0.00–2.34)
C73	Thyroid	[0.0, 0.5)	0	1	0.00 (0.00–2.74)
		[0.5, 1.0)	0	1	0.00 (0.00–3.05)
		[1.0, 2.0)	0	2	0.00 (0.00–1.76)
		[2.0, 3.0)	1	2	0.59 (0.01–3.28)
		[3.0, 5.0)	4	2	1.70 (0.46–4.35)
		[5.0, 10.0]	3	2	1.59 (0.33–4.64)
C76, C80	Unspecified site	[0.0, 0.5)	4	1	3.99 (1.09–10.22)
		[0.5, 1.0)	2	0	4.50 (0.55–16.26)
		[1.0, 2.0)	1	1	2.00 (0.05–11.17)
		[2.0, 3.0)	1	0	2.97 (0.08–16.55)
		[3.0, 5.0)	0	0	0.00 (0–8.55)
		[5.0, 10.0]	2	0	5.97 (0.72–21.56)
C81–C88	Lymphoma	[0.0, 0.5)	8	3	2.61 (1.12–5.13)
		[0.5, 1.0)	2	3	0.79 (0.10–2.84)
		[1.0, 2.0)	4	4	0.98 (0.27–2.51)
		[2.0, 3.0)	2	3	0.63 (0.08–2.28)
		[3.0, 5.0)	4	4	0.91 (0.25–2.34)
		[5.0, 10.0]	3	3	0.87 (0.18–2.53)
C90	Multiple myeloma	[0.0, 0.5)	3	1	2.50 (0.52–7.32)
		[0.5, 1.0)	3	1	3.12 (0.64–9.11)
		[1.0, 2.0)	2	1	1.36 (0.16–4.92)
		[2.0, 3.0)	0	1	0.00 (0.00–3.58)
		[3.0, 5.0)	0	1	0.00 (0.00–3.06)
		[5.0, 10.0]	3	1	4.24 (0.87–12.39)
C91–C96	Leukemia	[0.0, 0.5)	1	3	0.36 (0.01–2.01)
		[0.5, 1.0)	2	2	0.90 (0.11–3.24)
		[1.0, 2.0)	3	3	0.86 (0.18–2.51)
		[2.0, 3.0)	5	3	1.92 (0.62–4.49)
		[3.0, 5.0)	4	3	1.18 (0.32–3.02)
		[5.0, 10.0]	3	2	1.24 (0.26–3.64)

* All primary malignant neoplasms except non-melanoma skin cancers (C00–C43, C45–C76, and C80–C96 according to the ICD-10).

## Data Availability

We complied with all relevant ethical regulations. The data analyzed in this study were obtained from the PLCR and are available upon reasonable request by contacting the PLCR at krn@pib-nio.pl and subject to ethical approvals in place and material transfer agreements.

## Supplementary Materials

The following supporting information can be downloaded at: https://www.mdpi.com/article/10.3390/cancers15174315/s1, Table S1. Analysis by cancer site in the PolSCa study is based on the International Statistical Classification of Diseases and Related Health Problems 10th revision (ICD-10); Table S2. Characteristics of the study population; Table S3. Mechanism of suicide among patients with cancer by sex—Poland, 2009–2019.
