# Supplementary material for: Suicide after a Diagnosis of Cancer: Follow-Up of 1.4 Million Individuals, 2009–2019
Source: Cancers (Basel). 2023 Aug 29;15(17):4315. doi: 10.3390/cancers15174315 (PMC10486959; doi:10.3390/cancers15174315)
Supplement: Supplementary file 1 [file cancers-15-04315-s001.zip › cancers-2557713-supplementary.pdf]

Table S1. Analysis by cancer site in the PolSCa study is based on the International Statistical Classification of Diseases and Related Health Problems 10th revision (ICD-10). Some site codes were combined to provide data comparable with the previous literature. For the reader's convenience, all sites appearing under the specific ICD-10 codes are listed under one collective name - label.

ICD-10 code	Sites included	Label
**C00-C14 + C30**	Lip, oral cavity, pharynx, nasal cavity, and middle ear	Head and neck
**C15**	Esophagus	Esophagus
**C16**	Stomach	Stomach
**C17**	Small intestine	Small intestine
**C18-C20**	Colon, rectosigmoid junction, rectum	Colorectum
**C21**	Anus and anal canal	Anus
**C22-24**	Liver, intrahepatic bile ducts, gallbladder, biliary tract	Liver and gallbladder
**C25**	Pancreas	Pancreas
**C32**	Larynx	Larynx
**C33**	Trachea	Trachea
**C34**	Bronchus and lung	Lung
**C37**	Thymus	Thymus
**C38**	Heart, mediastinum, and pleura	Heart and pleura
**C40**	Bone and articular cartilage of limbs	Appendicular skeleton
**C41**	Bone and articular cartilage of other and unspecified sites	Axial skeleton
**C43**	Malignant melanoma of skin	Melanoma
**C45-C49**	Mesothelial and soft tissue	Soft tissues
**C50**	Breast	Breast
**C51-C52**	Vulva and vagina	Vulva and vagina
**C53**	Cervix uteri	Cervix uteri
**C54-C55**	Corpus uteri and uterus unspecified part	Corpus uteri
**C56**	Ovary	Ovary
**C60**	Penis	Penis
**C61**	Prostate	Prostate
**C62**	Testis	Testis
**C64**	Kidney, except renal pelvis	Kidney
**C65-C67**	Renal pelvis, ureter, and bladder	Bladder
**C69-C72**	Eye, brain, and other parts of central nervous system	Central nervous system
**C73**	Thyroid gland	Thyroid
**C74**	Adrenal gland	Adrenal gland
**C76, C80**	Other and ill-defined sites and unspecified sites	Unspecified site
**C81-C88**	Lymphoma	Lymphoma
**C90**	Multiple myeloma and malignant plasma cell neoplasms	Multiple myeloma
**C91-C96**	Leukemia	Leukemia

Table S2. Characteristics of the study population.

ICD-10	Site	Number of persons under follow-up			Person-years under risk of suicide			Age at diagnosis, mean (SD)			Number of suicides		
		Overall	Men	Women	Overall	Men	Women	Overall	Men	Women	Overall	Men	Women
	**All cancers***	1 426 661	717 144	709 517	3 627 793	1 622 638	2 005 155	64.1 (12.1)	65.1 (12.2)	63.0 (13.8)	830	683	147
C00-C14 + C30	Head and neck	43 136	30 876	12 260	95 596	63 816	31 780	61.8 (11.9)	61.1 (11.0)	63.5 (13.7)	49	47	2
C15	Esophagus	13 799	10 843	2 956	13 494	10 099	3 395	64.2 (10.6)	63.4 (10.0)	66.9 (12.1)	11	10	1
C16	Stomach	53 144	34 001	19 143	80 512	49 036	31 476	67.0 (12.1)	66.4 (11.5)	67.9 (13.1)	49	46	3
C17	Small intestine	3 212	1 657	1 555	7 962	4 093	3 869	63.4 (12.8)	62.3 (12.5)	64.7 (13.1)	2	2	0
C18-C20	Colorectum	178 267	99 329	78 938	461 416	250 212	211 205	67.6 (11.4)	67.1 (10.9)	68.3 (12.0)	134	121	13
C21	Anus	2 785	888	1 897	7 290	2 008	5 282	64.8 (12.2)	62.9 (12.5)	65.7 (12.0)	2	1	1
C22-24	Liver and gallbladder	28 362	12 693	15 669	32 707	14 544	18 163	67.7 (11.6)	66.0 (11.3)	69.1 (11.6)	8	7	1
C25	Pancreas	33 440	16 477	16 963	29 050	13 838	15 212	66.5 (11.4)	64.6 (10.8)	68.4 (11.6)	10	9	1
C32	Larynx	23 362	20 238	3 124	64 060	54 629	9 431	63.0 (9.3)	63.1 (9.3)	62.8 (9.5)	28	24	4
C33	Trachea	308	185	123	475	248	227	62.9 (11.7)	63.4 (10.8)	62.2 (12.9)	0	0	0
C34	Lung	210 584	140 184	70 400	265 661	167 877	97 784	66.0 (9.5)	66.2 (9.4)	65.7 (9.7)	100	89	11
C37	Thymus	790	373	417	2 341	1 100	1 241	57.0 (14.1)	55.4 (14.3)	58.4 (13.7)	0	0	0
C38	Heart and pleura	1 428	846	582	2 261	1 283	978	63.5 (16.2)	61.7 (16.5)	66.1 (15.4)	2	2	0
C40	Bone – limbs	1 472	823	649	5 044	2 682	2 362	45.4 (20.7)	43.4 (20.2)	48.0 (21.1)	0	0	0
C41	Bone – axial skeleton	1 770	924	846	4 691	2 219	2 472	53.0 (19.7)	52.8 (19.2)	53.3 (20.3)	1	1	0
C43	Melanoma	34 653	16 172	18 481	107 060	46 248	60 812	60.1 (16.4)	61.1 (15.3)	59.3 (17.3)	18	17	1
C45-C49	Soft tissues	13 272	7 162	6 110	30 647	15 470	15 177	60.4 (16.3)	59.9 (16.3)	60.9 (16.4)	6	6	0
C50	Breast	187 919	1 371	186 548	680 682	4 053	676 629	60.6 (13.0)	65.9 (11.8)	60.5 (13.0)	48	1	47
C51-C52	Vulva and vagina	6 433	-	6 433	15 393	-	15 393	70.4 (12.4)	-	70.4 (12.4)	3	-	3

ICD-10	Site	Number of persons under follow-up			Person-years under risk of suicide			Age at diagnosis, mean (SD)			Number of suicides		
		Overall	Men	Women	Overall	Men	Women	Overall	Men	Women	Overall	Men	Women
C53	Cervix uteri	28 903	-	28 903	90 365	-	90 365	57.1 (13.7)	-	57.1 (13.7)	10	-	10
C54-C55	Corpus uteri	61 037	-	61 037	215 944	-	215 944	64.1 (10.8)	-	64.1 (10.8)	12	-	12
C56	Ovary	37 976	-	37 976	102 438	-	102 438	60.1 (13.5)	-	60.1 (13.5)	8	-	8
C60	Penis	2 549	2 549	-	6 929	6 929	-	64.8 (12.7)	64.8 (12.7)	-	2	2	-
C61	Prostate	142 079	142 079	-	432 193	432 193	-	69.0 (8.4)	69.0 (8.4)	-	133	133	-
C62	Testis	12 193	12 193	-	51 221	51 221	-	34.6 (11.7)	34.6 (11.7)	-	18	18	-
C64	Kidney	51 349	30 915	20 434	155 159	89 332	65 826	63.9 (11.6)	63.0 (11.2)	65.4 (11.9)	32	27	5
C65-C67	Bladder	74 785	56 193	18 592	206 075	153 743	52 332	68.5 (10.9)	68.5 (10.7)	68.3 (11.5)	63	61	2
C69-C72	Central nervous system	32 663	16 364	16 299	65 452	31 039	34 413	58.6 (16.1)	57.1 (15.8)	60.2 (16.3)	11	9	2
C73	Thyroid gland	34 448	5 800	28 648	123 578	19 403	104 176	49.9 (15.6)	51.9 (15.8)	49.5 (15.5)	8	2	6
C74	Adrenal gland	1 072	479	593	2 935	1 124	1 811	59.0 (15.2)	59.0 (15.0)	58.9 (15.5)	0	0	0
C76, C80	Unspecified site	16 663	8 205	8 458	16 217	7 556	8 662	68.3 (12.8)	66.2 (12.2)	70.5 (13.1)	10	8	2
C81-C88	Lymphoma	38 841	19 466	19 375	119 243	58 128	61 115	58.3 (18.4)	57.5 (17.9)	59.1 (18.8)	23	14	9
C90	Multiple myeloma	15 477	7 294	8 183	36 408	16 606	19 802	66.9 (11.1)	65.8 (11.2)	67.9 (10.9)	11	9	2
C91-C96	Leukemia	32 611	17 957	14 654	85 566	46 764	38 802	64.3 (15.5)	63.1 (15.4)	65.9 (15.4)	18	17	1

* All primary malignant neoplasms except non-melanoma skin cancers (C00-C43, C45-C76, C80-C96 according to the ICD-10).

Table S3. Mechanism of suicide among patients with cancer by sex – Poland, 2009-2019.

ICD 10 code and mechanism of suicide	Overall	Men	Women
X60. Intentional self-poisoning by and exposure to nonopioid analgesics, antipyretics and antirheumatics	0.11%	0.00%	0.63%
X61. Intentional self-poisoning by and exposure to antiepileptic, sedative-hypnotic, antiparkinsonism and psychotropic drugs, not elsewhere classified	0.33%	0.26%	0.63%
X64. Intentional self-poisoning by and exposure to other and unspecified drugs, medicaments and biological substances	0.65%	0.00%	3.75%
X65. Intentional self-poisoning by and exposure to alcohol	2.94%	3.16%	1.88%
X67. Intentional self-poisoning by and exposure to carbon monoxide and other gases and vapours	0.22%	0.13%	0.63%
X69. Intentional self-poisoning by and exposure to other and unspecified chemicals and noxious substances	0.11%	0.13%	0.00%
X70. Intentional self-harm by hanging, strangulation and suffocation	84.55%	86.69%	74.38%
X71. Intentional self-harm by drowning and submersion	0.87%	0.66%	1.88%
X72. Intentional self-harm by handgun discharge	0.76%	0.92%	0.00%
X73. Intentional self-harm by rifle, shotgun and larger firearm discharge	0.44%	0.40%	0.63%
X74. Intentional self-harm by other and unspecified firearm discharge	0.44%	0.53%	0.00%
X75. Intentional self-harm by explosive material	0.11%	0.13%	0.00%
X78. Intentional self-harm by sharp object	2.39%	2.24%	3.13%
X79. Intentional self-harm by blunt object	0.11%	0.00%	0.63%
X80. Intentional self-harm by jumping from a high place	4.90%	4.08%	8.75%
X81. Intentional self-harm by jumping or lying before moving object	0.44%	0.13%	1.88%
X83. Intentional self-harm by other specified means	0.22%	0.13%	0.63%
X84. Intentional self-harm by unspecified means	0.44%	0.40%	0.63%
